# Tripotassium (bis­{[bis­(carboxyl­atometh­yl)amino]­meth­yl}phosphinato)cuprate(II) dihydrate

**DOI:** 10.1107/S1600536811053608

**Published:** 2011-12-21

**Authors:** Liyan Liu, Rui Zhang, Ping Fan, Zhan Yu, Xiangdong Zhang

**Affiliations:** a College of Chemistry and Biology, Shenyang Normal University, Shenyang, Liaoning 110000, People’s Republic of China; b College of Chemistry, Liaoning University, Shenyang, Liaoning 110036, People’s Republic of China

## Abstract

In the title compound, K_3_[Cu(C_10_H_12_N_2_O_10_P)]·2H_2_O, the Cu^II^ ion, one potassium cation and a P atom are situated on a twofold rotation axis. The Cu^II^ ion is coordinated by two N and four O atoms from one bis­{[bis­(carboxyl­atometh­yl)amino]­meth­yl}phosphinate ligand in a distorted octa­hedral coordination geometry. The two crystallographically independent potassium ions exhibit different coordination environments. The potassium ion in a general position is hepta­coordinated by five carboxyl­ate O atoms, one phosphinate O atom and one water mol­ecule [K—O = 2.718 (3)–3.040 (3) Å], and the potassium ion situated on the twofold rotation axis is hexa­coordinated by four carboxyl­ate O atoms and two water mol­ecules [K—O = 2.618 (3)–2.771 (3) Å]. The water mol­ecules are also involved in formation of inter­molecular O—H⋯O hydrogen bonds.

## Related literature

For details of the synthesis of the ligand, see: Varga (1997 ▶); Tircsó *et al.* (2007) ▶. For the isotypic compound with Co(II), see: Xu *et al.* (2001 ▶).
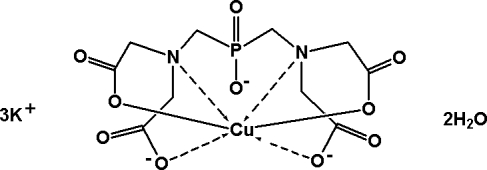


## Experimental

### 

#### Crystal data

K_3_[Cu(C_10_H_12_N_2_O_10_P)]·2H_2_O
*M* *_r_* = 568.06
Orthorhombic, 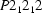

*a* = 11.880 (7) Å
*b* = 8.332 (5) Å
*c* = 9.681 (6) Å
*V* = 958.2 (10) Å^3^
*Z* = 2
Mo *K*α radiation
μ = 1.94 mm^−1^
*T* = 273 K
0.25 × 0.20 × 0.15 mm

#### Data collection

Bruker SMART CCD area-detector diffractometer
Absorption correction: multi-scan (*SADABS*; Sheldrick, 2000 ▶) *T*_min_ = 0.643, *T*_max_ = 0.760
3814 measured reflections
1686 independent reflections
1553 reflections with *I* > 2σ(*I*)
*R*_int_ = 0.041

#### Refinement

*R*[*F* ^2^ > 2σ(*F* ^2^)] = 0.025
*wR*(*F* ^2^) = 0.065
*S* = 1.07
1686 reflections
141 parameters
2 restraints
H atoms treated by a mixture of independent and constrained refinement
Δρ_max_ = 0.42 e Å^−3^
Δρ_min_ = −0.27 e Å^−3^
Absolute structure: Flack (1983 ▶), 671 Friedel pairs
Flack parameter: −0.016 (19)

### 

Data collection: *SMART* (Bruker, 2001 ▶); cell refinement: *SAINT* (Bruker, 2001 ▶); data reduction: *SAINT*; program(s) used to solve structure: *SHELXS97* (Sheldrick, 2008 ▶); program(s) used to refine structure: *SHELXL97* (Sheldrick, 2008 ▶); molecular graphics: *SHELXTL* (Sheldrick, 2008 ▶); software used to prepare material for publication: *SHELXL97*, *PLATON* (Spek, 2009 ▶) and *WinGX* (Farrugia, 1999 ▶).

## Supplementary Material

Crystal structure: contains datablock(s) I, global. DOI: 10.1107/S1600536811053608/cv5214sup1.cif

Structure factors: contains datablock(s) I. DOI: 10.1107/S1600536811053608/cv5214Isup2.hkl

Additional supplementary materials:  crystallographic information; 3D view; checkCIF report

## Figures and Tables

**Table 1:** Hydrogen-bond geometry (Å, °)

*D*—H⋯*A*	*D*—H	H⋯*A*	*D*⋯*A*	*D*—H⋯*A*
O6—H6*A*⋯O5^i^	0.93 (2)	1.75 (4)	2.682 (4)	173 (4)
O6—H6*B*⋯O1^ii^	0.94 (2)	2.02 (5)	2.860 (4)	148 (4)

Symmetry codes: (i) 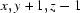; (ii) 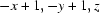.

**Fig. 1.**
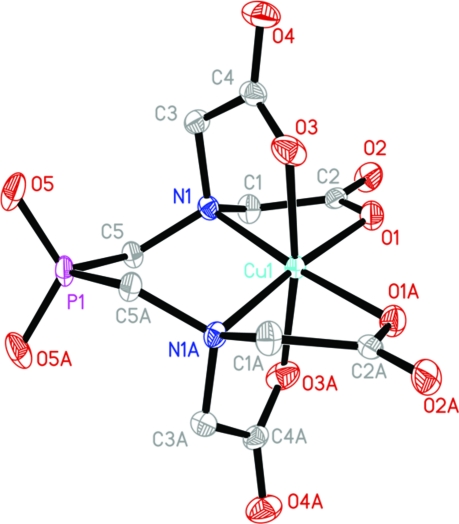
A portion of the crystal structure of (I) showing a coordination environment of CuII, atomic numbering and 50% probability displacement ellipsoids [symmetry code: (A) 1 - x, -y, z]. H atoms omitted for clarity.

**Fig. 2.**
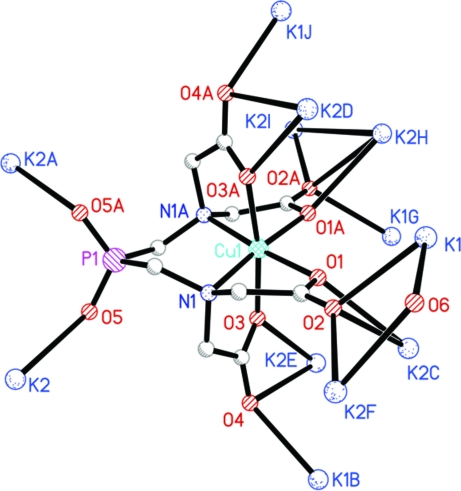
A portion of the crystal structure of (I) showing the positions of K+ [symmetry code: (A) 1 - x,-y, z; (B) 1/2 + x,1/2 - y, -z; (C) x,y, -1 + z; (D) -1/2 + x,1/2 - y, 1 - z; (E) 3/2 - x,-1/2 + y,1 - z; (F) 3/2 - x,1/2 + y, 1 - z; (G) x,-1 + y, z; (H) 1 - x,-y,-1 + z; (I) -1/2 + x,-1/2 - y,1 - z; (J) -1/2 + x,1/2 - y,-z]. O6 belongs to the water molecule. H atoms omitted for clarity.

## supplementary crystallographic information

### Comment

The most famous chelating ligands are aminopolycarboxylic acids such as the ethylenediaminetetraacetic acid (edta). In the current continuing quest for new chelating ligands, some derivatives of edta, which have both similar chelating properties as edta and special chemical fragments, are discovered. Bis{[bis(carboxymethyl)amino]methyl}phosphinic acid (H_5_XT) is a good example which could be structurally looked as two equal half of edta connected by a phosphinate group. Previous research (Xu *et al.*, 2001) has demonstrated that XT^5-^ is able to form stable complexes with rare earth metal and cobalt ions. Herewith we present the crystal structure of the title compound (I).

In (I) (Fig. 1), the Cu^II^ ion exhibits a distorted octahedral coordination geometry, where two N and two carboxylate O atoms located at the equatorial positions. Other two carboxylate O atoms occupy the axial positions. The Cu^II^ ion, one potassium cation and a P atom are situated on a twofold axis. Two types of potassium ions with different coodination circumstances are distributed in the title complex (Fig. 2). K1 is hexacoordinated by four carboxylate O atoms and two water molecules; while K2 is heptacoordinated by five carboxylate O atoms, one phosphinate O atom and one water molecule. The bond distances and angles in the title coupound agree well with the corresponding bond distances and angles reported in related [Co(II)XT]^3-^ complex (Xu *et al.*, 2001).

### Experimental

The ligand, bis{[bis(carboxymethyl)amino]methyl}phosphinic acid(XT), was synthesized according to the known procedure (Varga, 1997; Tircsó *et al.*, 2007).

The title complex was simply synthesized by mixing 0.4027 g XT, 0.1774 g CuCl_2_ and 5 ml water in a small beaker with sufficient stirring. When the solution became clear, KOH was used to adjust the pH value to 8. Then the beaker was transferred to a closed container of methanol. After methanol vapor diffusion for one week, blue transparent crystals were observed from the solution.

### Refinement

C-bound H atoms were geometrically positioned [C—H 0.97 Å], and refined as riding, with *U*_iso_(H) = 1.2*U*_eq_(C). O-bound H atoms were located in a difference Fourier map, and refined with restraint O—H = 0.93 (2) Å, and with *U*_iso_(H) = 1.5 *U*_eq_(O).

### Crystal data

**Table:**

K_3_[Cu(C_10_H_12_N_2_O_10_P)]·2H_2_O	*F*(000) = 574
*M**_r_* = 568.06	*D*_x_ = 1.969 Mg m^−^^3^
Orthorhombic, *P*2_1_2_1_2	Mo *K*α radiation, λ = 0.71073 Å
Hall symbol: P 2 2ab	Cell parameters from 376 reflections
*a* = 11.880 (7) Å	θ = 2.6–22.8°
*b* = 8.332 (5) Å	µ = 1.94 mm^−^^1^
*c* = 9.681 (6) Å	*T* = 273 K
*V* = 958.2 (10) Å^3^	Block, blue
*Z* = 2	0.25 × 0.20 × 0.15 mm

### Data collection

**Table:**

Bruker SMART CCD area-detector diffractometer	1686 independent reflections
Radiation source: fine-focus sealed tube	1553 reflections with *I* > 2σ(*I*)
graphite	*R*_int_ = 0.041
phi and ω scans	θ_max_ = 25.0°, θ_min_ = 2.1°
Absorption correction: multi-scan (*SADABS*; Sheldrick, 2000)	*h* = −14→7
*T*_min_ = 0.643, *T*_max_ = 0.760	*k* = −9→9
3814 measured reflections	*l* = −11→11

### Refinement

**Table:**

Refinement on *F*^2^	Secondary atom site location: difference Fourier map
Least-squares matrix: full	Hydrogen site location: inferred from neighbouring sites
*R*[*F*^2^ > 2σ(*F*^2^)] = 0.025	H atoms treated by a mixture of independent and constrained refinement
*wR*(*F*^2^) = 0.065	*w* = 1/[σ^2^(*F*_o_^2^) + (0.0309*P*)^2^ + 0.1108*P*] where *P* = (*F*_o_^2^ + 2*F*_c_^2^)/3
*S* = 1.07	(Δ/σ)_max_ < 0.001
1686 reflections	Δρ_max_ = 0.42 e Å^−^^3^
141 parameters	Δρ_min_ = −0.27 e Å^−^^3^
2 restraints	Absolute structure: Flack (1983), 671 Friedel pairs
Primary atom site location: structure-invariant direct methods	Flack parameter: −0.016 (19)

### Special details

**Table:**

Geometry. All e.s.d.'s (except the e.s.d. in the dihedral angle between two l.s. planes) are estimated using the full covariance matrix. The cell e.s.d.'s are taken into account individually in the estimation of e.s.d.'s in distances, angles and torsion angles; correlations between e.s.d.'s in cell parameters are only used when they are defined by crystal symmetry. An approximate (isotropic) treatment of cell e.s.d.'s is used for estimating e.s.d.'s involving l.s. planes.
Refinement. Refinement of *F*^2^ against ALL reflections. The weighted *R*-factor *wR* and goodness of fit *S* are based on *F*^2^, conventional *R*-factors *R* are based on *F*, with *F* set to zero for negative *F*^2^. The threshold expression of *F*^2^ > σ(*F*^2^) is used only for calculating *R*-factors(gt) *etc*. and is not relevant to the choice of reflections for refinement. *R*-factors based on *F*^2^ are statistically about twice as large as those based on *F*, and *R*- factors based on ALL data will be even larger.

### Fractional atomic coordinates and isotropic or equivalent isotropic displacement parameters (Å^2^)

**Table:**

	*x*	*y*	*z*	*U*_iso_*/*U*_eq_	
Cu1	0.5000	0.0000	0.24820 (5)	0.02067 (13)	
N1	0.5819 (2)	0.1559 (3)	0.3846 (2)	0.0241 (5)	
O1	0.56779 (19)	0.1530 (3)	0.1064 (2)	0.0350 (5)	
O2	0.6252 (2)	0.4027 (3)	0.0863 (2)	0.0439 (7)	
O3	0.65208 (19)	−0.1264 (3)	0.2638 (3)	0.0431 (6)	
O4	0.83096 (19)	−0.0736 (3)	0.3018 (3)	0.0480 (7)	
O5	0.6061 (2)	−0.0298 (3)	0.7037 (2)	0.0451 (7)	
O6	0.6136 (3)	0.7735 (4)	−0.0772 (3)	0.0451 (7)	
K1	0.5000	0.5000	−0.14025 (10)	0.0354 (2)	
K2	0.72836 (6)	0.15198 (9)	0.89192 (7)	0.03197 (18)	
P1	0.5000	0.0000	0.62460 (10)	0.0294 (3)	
C1	0.5802 (3)	0.3100 (4)	0.3092 (3)	0.0309 (8)	
H1A	0.6403	0.3779	0.3435	0.037*	
H1B	0.5094	0.3641	0.3273	0.037*	
C2	0.5938 (3)	0.2890 (4)	0.1561 (4)	0.0279 (7)	
C3	0.6992 (3)	0.0996 (4)	0.4033 (4)	0.0314 (7)	
H3A	0.7104	0.0719	0.4997	0.038*	
H3B	0.7501	0.1871	0.3816	0.038*	
C4	0.7300 (3)	−0.0441 (4)	0.3151 (3)	0.0300 (8)	
C5	0.5181 (3)	0.1745 (4)	0.5159 (3)	0.0279 (7)	
H5B	0.4439	0.2150	0.4930	0.033*	
H5A	0.5554	0.2565	0.5703	0.033*	
H6A	0.613 (3)	0.835 (5)	−0.158 (3)	0.059 (13)*	
H6B	0.569 (4)	0.835 (6)	−0.017 (5)	0.10 (2)*	

### Atomic displacement parameters (Å^2^)

**Table:**

	*U*^11^	*U*^22^	*U*^33^	*U*^12^	*U*^13^	*U*^23^
Cu1	0.0246 (2)	0.0185 (2)	0.0189 (2)	−0.0019 (2)	0.000	0.000
N1	0.0280 (12)	0.0227 (13)	0.0216 (13)	0.0016 (11)	0.0028 (11)	0.0039 (13)
O1	0.0404 (13)	0.0380 (13)	0.0266 (12)	−0.0059 (11)	0.0010 (10)	−0.0022 (12)
O2	0.0466 (15)	0.0481 (16)	0.0369 (14)	−0.0133 (12)	−0.0063 (11)	0.0228 (13)
O3	0.0383 (12)	0.0399 (15)	0.0510 (16)	−0.0032 (12)	0.0113 (12)	−0.0146 (13)
O4	0.0315 (13)	0.0526 (16)	0.0599 (17)	0.0113 (13)	0.0084 (12)	0.0057 (14)
O5	0.0594 (15)	0.0483 (18)	0.0277 (11)	−0.0152 (14)	−0.0179 (11)	0.0126 (11)
O6	0.0528 (17)	0.0480 (16)	0.0346 (16)	0.0074 (13)	0.0055 (13)	0.0145 (13)
K1	0.0282 (4)	0.0441 (6)	0.0339 (5)	0.0023 (6)	0.000	0.000
K2	0.0348 (3)	0.0312 (4)	0.0299 (4)	0.0003 (3)	0.0005 (3)	−0.0034 (3)
P1	0.0408 (6)	0.0328 (6)	0.0145 (5)	−0.0103 (6)	0.000	0.000
C1	0.044 (2)	0.0214 (18)	0.0277 (17)	−0.0030 (14)	0.0002 (15)	0.0030 (14)
C2	0.0219 (15)	0.036 (2)	0.0260 (17)	−0.0021 (15)	−0.0033 (13)	0.0058 (16)
C3	0.0267 (16)	0.0380 (19)	0.0296 (18)	−0.0031 (14)	−0.0025 (13)	0.0026 (16)
C4	0.0267 (15)	0.033 (2)	0.0299 (16)	0.0030 (14)	0.0044 (14)	0.0098 (13)
C5	0.0336 (18)	0.0290 (16)	0.0209 (14)	−0.0030 (14)	0.0033 (13)	−0.0010 (13)

### Geometric parameters (Å, °)

**Table:**

Cu1—O1^i^	2.039 (3)	K1—O4^vi^	2.618 (3)
Cu1—O1	2.039 (3)	K1—O4^vii^	2.618 (3)
Cu1—N1	2.092 (3)	K1—O6^viii^	2.718 (3)
Cu1—N1^i^	2.092 (3)	K1—O2^viii^	2.771 (3)
Cu1—O3^i^	2.097 (3)	K2—O2^iv^	2.717 (3)
Cu1—O3	2.097 (3)	K2—O3^iii^	2.775 (3)
N1—C1	1.478 (4)	K2—O6^iv^	2.788 (3)
N1—C3	1.482 (4)	K2—O1^ix^	2.819 (3)
N1—C5	1.488 (4)	K2—O4^iii^	3.040 (3)
O1—C2	1.270 (4)	K2—O2^ix^	3.067 (3)
O1—K2^ii^	2.819 (3)	K2—C2^ix^	3.225 (4)
O2—C2	1.222 (4)	K2—C4^iii^	3.267 (4)
O2—K2^iii^	2.717 (3)	P1—O5^i^	1.496 (2)
O2—K1	2.771 (3)	P1—C5^i^	1.807 (3)
O2—K2^ii^	3.067 (3)	P1—C5	1.807 (3)
O3—C4	1.255 (4)	C1—C2	1.501 (5)
O3—K2^iv^	2.775 (3)	C1—H1A	0.9700
O4—C4	1.231 (4)	C1—H1B	0.9700
O4—K1^v^	2.618 (3)	C2—K2^ii^	3.225 (4)
O4—K2^iv^	3.040 (3)	C3—C4	1.515 (5)
O5—P1	1.496 (2)	C3—H3A	0.9700
O5—K2	2.779 (3)	C3—H3B	0.9700
O6—K1	2.718 (3)	C4—K2^iv^	3.267 (4)
O6—K2^iii^	2.788 (3)	C5—H5B	0.9700
O6—H6A	0.932 (19)	C5—H5A	0.9700
O6—H6B	0.94 (2)		
			
O1^i^—Cu1—O1	95.35 (14)	O3^iii^—K2—O1^ix^	138.11 (8)
O1^i^—Cu1—N1	175.30 (10)	O5—K2—O1^ix^	97.50 (9)
O1—Cu1—N1	81.57 (10)	O6^iv^—K2—O1^ix^	88.91 (9)
O1^i^—Cu1—N1^i^	81.57 (10)	O2^iv^—K2—O4^iii^	140.46 (8)
O1—Cu1—N1^i^	175.30 (10)	O3^iii^—K2—O4^iii^	44.19 (7)
N1—Cu1—N1^i^	101.72 (14)	O5—K2—O4^iii^	83.36 (8)
O1^i^—Cu1—O3^i^	91.27 (10)	O6^iv^—K2—O4^iii^	106.25 (9)
O1—Cu1—O3^i^	94.30 (10)	O1^ix^—K2—O4^iii^	107.17 (8)
N1—Cu1—O3^i^	92.50 (10)	O2^iv^—K2—O2^ix^	136.78 (5)
N1^i^—Cu1—O3^i^	82.27 (10)	O3^iii^—K2—O2^ix^	94.85 (8)
O1^i^—Cu1—O3	94.30 (10)	O5—K2—O2^ix^	124.36 (8)
O1—Cu1—O3	91.27 (10)	O6^iv^—K2—O2^ix^	68.10 (9)
N1—Cu1—O3	82.27 (10)	O1^ix^—K2—O2^ix^	43.61 (7)
N1^i^—Cu1—O3	92.50 (10)	O4^iii^—K2—O2^ix^	76.91 (8)
O3^i^—Cu1—O3	171.73 (14)	O2^iv^—K2—C2^ix^	121.76 (9)
C1—N1—C3	110.4 (3)	O3^iii^—K2—C2^ix^	116.68 (9)
C1—N1—C5	108.9 (3)	O5—K2—C2^ix^	116.98 (9)
C3—N1—C5	114.1 (2)	O6^iv^—K2—C2^ix^	72.25 (9)
C1—N1—Cu1	102.74 (19)	O1^ix^—K2—C2^ix^	23.02 (8)
C3—N1—Cu1	108.5 (2)	O4^iii^—K2—C2^ix^	96.20 (9)
C5—N1—Cu1	111.52 (19)	O2^ix^—K2—C2^ix^	22.22 (7)
C2—O1—Cu1	113.5 (2)	O2^iv^—K2—C4^iii^	122.90 (9)
C2—O1—K2^ii^	96.7 (2)	O3^iii^—K2—C4^iii^	22.10 (7)
Cu1—O1—K2^ii^	139.57 (12)	O5—K2—C4^iii^	95.70 (9)
C2—O2—K2^iii^	138.2 (2)	O6^iv^—K2—C4^iii^	90.64 (9)
C2—O2—K1	120.0 (2)	O1^ix^—K2—C4^iii^	123.49 (8)
K2^iii^—O2—K1	100.46 (8)	O4^iii^—K2—C4^iii^	22.13 (7)
C2—O2—K2^ii^	86.2 (2)	O2^ix^—K2—C4^iii^	84.76 (8)
K2^iii^—O2—K2^ii^	108.21 (9)	C2^ix^—K2—C4^iii^	106.68 (9)
K1—O2—K2^ii^	85.89 (8)	O2^iv^—K2—K1^ix^	176.86 (6)
C4—O3—Cu1	113.0 (2)	O3^iii^—K2—K1^ix^	79.73 (7)
C4—O3—K2^iv^	101.58 (19)	O5—K2—K1^ix^	89.39 (7)
Cu1—O3—K2^iv^	137.40 (12)	O6^iv^—K2—K1^ix^	104.17 (8)
C4—O4—K1^v^	139.7 (2)	O1^ix^—K2—K1^ix^	66.08 (6)
C4—O4—K2^iv^	89.3 (2)	O4^iii^—K2—K1^ix^	41.09 (5)
K1^v^—O4—K2^iv^	89.16 (9)	O2^ix^—K2—K1^ix^	43.94 (5)
P1—O5—K2	133.30 (16)	C2^ix^—K2—K1^ix^	57.76 (7)
K1—O6—K2^iii^	100.04 (10)	C4^iii^—K2—K1^ix^	59.39 (6)
K1—O6—H6A	106 (3)	O2^iv^—K2—K1^iv^	40.23 (5)
K2^iii^—O6—H6A	138 (3)	O3^iii^—K2—K1^iv^	96.76 (7)
K1—O6—H6B	109 (4)	O5—K2—K1^iv^	127.58 (7)
K2^iii^—O6—H6B	100 (3)	O6^iv^—K2—K1^iv^	39.37 (7)
H6A—O6—H6B	102 (4)	O1^ix^—K2—K1^iv^	95.67 (7)
O4^vi^—K1—O4^vii^	106.63 (14)	O4^iii^—K2—K1^iv^	138.99 (5)
O4^vi^—K1—O6	108.58 (9)	O2^ix^—K2—K1^iv^	99.24 (6)
O4^vii^—K1—O6	87.12 (9)	C2^ix^—K2—K1^iv^	91.93 (7)
O4^vi^—K1—O6^viii^	87.12 (9)	C4^iii^—K2—K1^iv^	117.81 (7)
O4^vii^—K1—O6^viii^	108.58 (9)	K1^ix^—K2—K1^iv^	141.64 (3)
O6—K1—O6^viii^	154.05 (12)	O2^iv^—K2—K2^xii^	125.03 (7)
O4^vi^—K1—O2	162.36 (8)	O3^iii^—K2—K2^xii^	66.12 (7)
O4^vii^—K1—O2	89.57 (9)	O5—K2—K2^xii^	146.49 (6)
O6—K1—O2	78.53 (9)	O6^iv^—K2—K2^xii^	46.87 (7)
O6^viii^—K1—O2	80.99 (9)	O1^ix^—K2—K2^xii^	75.11 (6)
O4^vi^—K1—O2^viii^	89.57 (9)	O4^iii^—K2—K2^xii^	68.44 (7)
O4^vii^—K1—O2^viii^	162.36 (8)	O2^ix^—K2—K2^xii^	33.39 (5)
O6—K1—O2^viii^	80.99 (9)	C2^ix^—K2—K2^xii^	52.12 (7)
O6^viii^—K1—O2^viii^	78.53 (9)	C4^iii^—K2—K2^xii^	64.45 (7)
O2—K1—O2^viii^	75.37 (12)	K1^ix^—K2—K2^xii^	57.52 (3)
O4^vi^—K1—K2^x^	49.75 (7)	K1^iv^—K2—K2^xii^	85.90 (3)
O4^vii^—K1—K2^x^	137.69 (7)	O5—P1—O5^i^	118.4 (2)
O6—K1—K2^x^	73.15 (8)	O5—P1—C5^i^	105.36 (15)
O6^viii^—K1—K2^x^	104.76 (8)	O5^i^—P1—C5^i^	109.36 (14)
O2—K1—K2^x^	121.11 (7)	O5—P1—C5	109.36 (14)
O2^viii^—K1—K2^x^	50.17 (6)	O5^i^—P1—C5	105.37 (15)
O4^vi^—K1—K2^ii^	137.69 (7)	C5^i^—P1—C5	108.8 (2)
O4^vii^—K1—K2^ii^	49.75 (7)	N1—C1—C2	112.7 (3)
O6—K1—K2^ii^	104.76 (8)	N1—C1—H1A	109.1
O6^viii^—K1—K2^ii^	73.15 (8)	C2—C1—H1A	109.1
O2—K1—K2^ii^	50.17 (6)	N1—C1—H1B	109.1
O2^viii^—K1—K2^ii^	121.11 (7)	C2—C1—H1B	109.1
K2^x^—K1—K2^ii^	171.03 (3)	H1A—C1—H1B	107.8
O4^vi^—K1—K2^iii^	148.97 (7)	O2—C2—O1	123.8 (3)
O4^vii^—K1—K2^iii^	79.86 (7)	O2—C2—C1	119.3 (3)
O6—K1—K2^iii^	40.59 (6)	O1—C2—C1	116.9 (3)
O6^viii^—K1—K2^iii^	120.23 (7)	O2—C2—K2^ii^	71.6 (2)
O2—K1—K2^iii^	39.30 (6)	O1—C2—K2^ii^	60.27 (17)
O2^viii^—K1—K2^iii^	82.64 (7)	C1—C2—K2^ii^	151.4 (2)
K2^x^—K1—K2^iii^	104.95 (4)	N1—C3—C4	114.1 (3)
K2^ii^—K1—K2^iii^	69.69 (4)	N1—C3—H3A	108.7
O4^vi^—K1—K2^xi^	79.86 (7)	C4—C3—H3A	108.7
O4^vii^—K1—K2^xi^	148.97 (7)	N1—C3—H3B	108.7
O6—K1—K2^xi^	120.23 (7)	C4—C3—H3B	108.7
O6^viii^—K1—K2^xi^	40.59 (6)	H3A—C3—H3B	107.6
O2—K1—K2^xi^	82.64 (7)	O4—C4—O3	124.7 (3)
O2^viii^—K1—K2^xi^	39.30 (6)	O4—C4—C3	116.8 (3)
K2^x^—K1—K2^xi^	69.69 (4)	O3—C4—C3	118.5 (3)
K2^ii^—K1—K2^xi^	104.95 (4)	O4—C4—K2^iv^	68.5 (2)
K2^iii^—K1—K2^xi^	110.52 (5)	O3—C4—K2^iv^	56.32 (16)
O2^iv^—K2—O3^iii^	102.89 (9)	C3—C4—K2^iv^	174.0 (2)
O2^iv^—K2—O5	88.23 (9)	N1—C5—P1	118.3 (2)
O3^iii^—K2—O5	105.91 (9)	N1—C5—H5B	107.7
O2^iv^—K2—O6^iv^	78.25 (9)	P1—C5—H5B	107.7
O3^iii^—K2—O6^iv^	76.29 (9)	N1—C5—H5A	107.7
O5—K2—O6^iv^	166.42 (9)	P1—C5—H5A	107.7
O2^iv^—K2—O1^ix^	112.22 (8)	H5B—C5—H5A	107.1
			
O1^i^—Cu1—N1—C1	79.0 (13)	K2^iii^—O2—K1—K2^ii^	107.77 (9)
O1—Cu1—N1—C1	29.69 (19)	C2—O2—K1—K2^iii^	169.1 (3)
N1^i^—Cu1—N1—C1	−146.9 (2)	K2^ii^—O2—K1—K2^iii^	−107.77 (9)
O3^i^—Cu1—N1—C1	−64.3 (2)	C2—O2—K1—K2^xi^	33.3 (3)
O3—Cu1—N1—C1	122.1 (2)	K2^iii^—O2—K1—K2^xi^	−135.71 (8)
O1^i^—Cu1—N1—C3	−38.0 (13)	K2^ii^—O2—K1—K2^xi^	116.52 (6)
O1—Cu1—N1—C3	−87.3 (2)	P1—O5—K2—O2^iv^	179.09 (18)
N1^i^—Cu1—N1—C3	96.1 (2)	P1—O5—K2—O3^iii^	−78.01 (19)
O3^i^—Cu1—N1—C3	178.8 (2)	P1—O5—K2—O6^iv^	−175.5 (3)
O3—Cu1—N1—C3	5.2 (2)	P1—O5—K2—O1^ix^	66.90 (19)
O1^i^—Cu1—N1—C5	−164.5 (12)	P1—O5—K2—O4^iii^	−39.62 (17)
O1—Cu1—N1—C5	146.2 (2)	P1—O5—K2—O2^ix^	29.5 (2)
N1^i^—Cu1—N1—C5	−30.36 (16)	P1—O5—K2—C2^ix^	54.0 (2)
O3^i^—Cu1—N1—C5	52.3 (2)	P1—O5—K2—C4^iii^	−58.02 (19)
O3—Cu1—N1—C5	−121.3 (2)	P1—O5—K2—K1^ix^	1.13 (17)
O1^i^—Cu1—O1—C2	162.8 (3)	P1—O5—K2—K1^iv^	170.04 (14)
N1—Cu1—O1—C2	−20.8 (2)	P1—O5—K2—K2^xii^	−7.4 (3)
N1^i^—Cu1—O1—C2	113.9 (12)	K2—O5—P1—O5^i^	−60.87 (14)
O3^i^—Cu1—O1—C2	71.1 (2)	K2—O5—P1—C5^i^	176.51 (17)
O3—Cu1—O1—C2	−102.8 (2)	K2—O5—P1—C5	59.7 (2)
O1^i^—Cu1—O1—K2^ii^	−62.18 (14)	C3—N1—C1—C2	79.8 (4)
N1—Cu1—O1—K2^ii^	114.25 (17)	C5—N1—C1—C2	−154.1 (3)
N1^i^—Cu1—O1—K2^ii^	−111.0 (12)	Cu1—N1—C1—C2	−35.8 (3)
O3^i^—Cu1—O1—K2^ii^	−153.86 (17)	K2^iii^—O2—C2—O1	−144.8 (3)
O3—Cu1—O1—K2^ii^	32.26 (16)	K1—O2—C2—O1	51.4 (4)
O1^i^—Cu1—O3—C4	161.4 (2)	K2^ii^—O2—C2—O1	−31.6 (3)
O1—Cu1—O3—C4	65.9 (2)	K2^iii^—O2—C2—C1	37.7 (5)
N1—Cu1—O3—C4	−15.4 (2)	K1—O2—C2—C1	−126.1 (3)
N1^i^—Cu1—O3—C4	−116.9 (2)	K2^ii^—O2—C2—C1	150.9 (3)
O3^i^—Cu1—O3—C4	−66.4 (2)	K2^iii^—O2—C2—K2^ii^	−113.2 (3)
O1^i^—Cu1—O3—K2^iv^	19.87 (17)	K1—O2—C2—K2^ii^	83.01 (17)
O1—Cu1—O3—K2^iv^	−75.59 (17)	Cu1—O1—C2—O2	−172.6 (3)
N1—Cu1—O3—K2^iv^	−156.91 (18)	K2^ii^—O1—C2—O2	34.9 (3)
N1^i^—Cu1—O3—K2^iv^	101.60 (17)	Cu1—O1—C2—C1	5.0 (4)
O3^i^—Cu1—O3—K2^iv^	152.06 (16)	K2^ii^—O1—C2—C1	−147.5 (3)
K2^iii^—O6—K1—O4^vi^	175.67 (8)	Cu1—O1—C2—K2^ii^	152.51 (19)
K2^iii^—O6—K1—O4^vii^	−77.75 (11)	N1—C1—C2—O2	−159.6 (3)
K2^iii^—O6—K1—O6^viii^	50.96 (7)	N1—C1—C2—O1	22.7 (4)
K2^iii^—O6—K1—O2	12.40 (8)	N1—C1—C2—K2^ii^	−53.8 (6)
K2^iii^—O6—K1—O2^viii^	89.18 (10)	C1—N1—C3—C4	−108.3 (3)
K2^iii^—O6—K1—K2^x^	140.16 (9)	C5—N1—C3—C4	128.6 (3)
K2^iii^—O6—K1—K2^ii^	−30.77 (9)	Cu1—N1—C3—C4	3.6 (3)
K2^iii^—O6—K1—K2^xi^	86.76 (10)	K1^v^—O4—C4—O3	92.5 (5)
C2—O2—K1—O4^vi^	40.5 (5)	K2^iv^—O4—C4—O3	4.6 (3)
K2^iii^—O2—K1—O4^vi^	−128.5 (3)	K1^v^—O4—C4—C3	−89.3 (4)
K2^ii^—O2—K1—O4^vi^	123.7 (3)	K2^iv^—O4—C4—C3	−177.2 (2)
C2—O2—K1—O4^vii^	−116.5 (3)	K1^v^—O4—C4—K2^iv^	87.9 (3)
K2^iii^—O2—K1—O4^vii^	74.40 (9)	Cu1—O3—C4—O4	−159.7 (3)
K2^ii^—O2—K1—O4^vii^	−33.37 (7)	K2^iv^—O3—C4—O4	−5.2 (4)
C2—O2—K1—O6	156.3 (3)	Cu1—O3—C4—C3	22.1 (3)
K2^iii^—O2—K1—O6	−12.74 (9)	K2^iv^—O3—C4—C3	176.7 (2)
K2^ii^—O2—K1—O6	−120.52 (9)	Cu1—O3—C4—K2^iv^	−154.5 (2)
C2—O2—K1—O6^viii^	−7.7 (3)	N1—C3—C4—O4	163.9 (3)
K2^iii^—O2—K1—O6^viii^	−176.70 (10)	N1—C3—C4—O3	−17.8 (4)
K2^ii^—O2—K1—O6^viii^	75.52 (8)	N1—C3—C4—K2^iv^	10 (2)
C2—O2—K1—O2^viii^	72.7 (3)	C1—N1—C5—P1	176.6 (2)
K2^iii^—O2—K1—O2^viii^	−96.30 (12)	C3—N1—C5—P1	−59.6 (3)
K2^ii^—O2—K1—O2^viii^	155.92 (11)	Cu1—N1—C5—P1	63.8 (3)
C2—O2—K1—K2^x^	94.2 (3)	O5—P1—C5—N1	78.5 (3)
K2^iii^—O2—K1—K2^x^	−74.84 (9)	O5^i^—P1—C5—N1	−153.3 (2)
K2^ii^—O2—K1—K2^x^	177.388 (15)	C5^i^—P1—C5—N1	−36.14 (18)
C2—O2—K1—K2^ii^	−83.2 (3)		

Symmetry codes: (i) −*x*+1, −*y*, *z*; (ii) *x*, *y*, *z*−1; (iii) −*x*+3/2, *y*+1/2, −*z*+1; (iv) −*x*+3/2, *y*−1/2, −*z*+1; (v) −*x*+3/2, *y*−1/2, −*z*; (vi) *x*−1/2, −*y*+1/2, −*z*; (vii) −*x*+3/2, *y*+1/2, −*z*; (viii) −*x*+1, −*y*+1, *z*; (ix) *x*, *y*, *z*+1; (x) −*x*+1, −*y*+1, *z*−1; (xi) *x*−1/2, −*y*+1/2, −*z*+1; (xii) −*x*+3/2, *y*+1/2, −*z*+2.

### Hydrogen-bond geometry (Å, °)

**Table:**

*D*—H···*A*	*D*—H	H···*A*	*D*···*A*	*D*—H···*A*
O6—H6A···O5^xiii^	0.93 (2)	1.75 (4)	2.682 (4)	173 (4)
O6—H6B···O1^viii^	0.94 (2)	2.02 (5)	2.860 (4)	148 (4)

Symmetry codes: (xiii) *x*, *y*+1, *z*−1; (viii) −*x*+1, −*y*+1, *z*.
